# Preparation of Erlotinib hydrochloride nanoparticles (anti-cancer drug) by RESS-C method and investigating the effective parameters

**DOI:** 10.1038/s41598-024-64477-8

**Published:** 2024-06-28

**Authors:** Majid Bazaei, Bizhan Honarvar, Nadia Esfandiari, Seyed Ali Sajadian, Zahra Arab Aboosadi

**Affiliations:** 1 Department of Chemical Engineering, Marvdasht Branch, Islamic Azad University, Marvdasht, Iran; 2 Department of Chemical Engineering, Faculty of Engineering, University of Kashan, Kashan, 87317-53153 Iran; 3 South Zagros Oil and Gas Production, National Iranian Oil Company, Shiraz, 7135717991 Iran

**Keywords:** Erlotinib hydrochloride, RESS-C method, Particle size, Pharmaceutical particles, Anticancer drug, Biomedical engineering, Chemical engineering, Cancer, Drug discovery, Chemistry

## Abstract

The size of the drug particles is one of the essential factors for the proper absorption of the drug compared to the dose of the drug. When particle size is decreased, drug uptake into the body increases. Recent studies have revealed that the rapid expansion of supercritical solution with cosolvent plays a significant role in preparing micron and submicron particles. This paper examines the preparation of Erlotinib hydrochloride nanoparticles using a supercritical solution through the cosolvent method for the first time. An examination of the parameters of temperature (318–338 K), pressures (15–25 MPa) and nozzle diameter (300–700 μm) was investigated by Box-Behnken design, and their respective effects on particle size revealed that the nozzle diameter has a more significant impact on particle size than the other parameters. The smallest particles were produced at temperature 338 K, pressure 20 MPa, and nozzle diameter 700 μm. Besides, the ERL nanoparticles were characterized using SEM, DLS, XRD, FTIR, and DSC analyses. Finally, the results showed that the average size of the ERL particles decreased from 31.6 μm to 200–1100 nm.

## Introduction

Cancers affect different parts of the body. A highly critical organ that can be affected by this deadly disease is the lung. This type of cancer is increasing worldwide, and many people are suffering from it^1^. Erlotinib hydrochloride (ERL), known as Tarceva, is a drug used to treat certain cancers. This drug prevents the growth of cancer cells and their spread in the body. ERL is used to treat lung cancer, and in some cases, pancreatic cancer^2^. ERL (C_22_H_23_N_3_O_4_.HCl) is a Biopharmaceutics Classification System (BCS) class II drug that is very slightly soluble in water and has low permeability. The maximum solubility of ERL at a pH of approximately two has been reported to be about 0.4 mg/ml^3^. The low solubility of pharmaceutical compounds in the body results in heightened drug usage, increased adverse effects, and diminished effectiveness^4^. Because of its very low solubility, ERL requires a high dose for better effectiveness. Production of micro/nanoparticles of pharmaceutical compounds with uniform morphology and size distribution is one of the approved methods to increase the solubility of these compounds. Therefore, the selection and design of an appropriate method for producing micro/nanoparticles of pharmaceutical compounds is one of the highly crucial research areas in the pharmaceutical industry^5–7^. Since one of the most prominent goals of pharmaceutical companies is to produce a product with high solubility and bioavailability, proper performance and absorption in the body, and minimizing side effects, various researches have been conducted on this issue, and the results reported by researchers It shows that reducing the particle size of a medicine improves its function and absorption in the body and reduces the side effects caused by its use^6,8–11^. There are various conventional methods to reduce the size of particles, including spray drying, grinding, and evaporation, among others. However, these methods have disadvantages such as a multi-stage production process, poor control over particle size, wide distribution of particles and quality change due to thermal effects, high consumption of solvents, and solvent removal. Investigations have shown that using supercritical fluids (SCFs) to prepare fine particles can be a good alternative to solve these disadvantages. These fluids are single-phase and have quasi-gas and quasi-liquid properties. They act similarly to liquids in terms of solubility and gases regarding permeability. As a result, their solubility power and selectivity can be increased under suitable temperature and pressure conditions^12–16^. Different SCFs, such as carbon dioxide (CO_2_), propane, water, methanol, ethanol, and ethane, have thus far been used to prepare fine particles. Nevertheless, among these SFCs, CO_2_ has received substantial attention for various reasons, including its low critical temperature, non-toxicity, non-flammability, availability, and reasonable price. With a critical pressure of 7.38 MPa and a critical temperature of 304.18 K, CO_2_ has suitable conditions for various processes^17–23^. Supercritical CO_2_ (SC-CO_2_) can be used as a solvent, e.g. the rapid expansion of supercritical solutions (RESS) or anti-solvent, e.g. supercritical antisolvent system (SAS), gas antisolvent system (GAS), aerosol solvent extraction system (ASES), and precipitation with compressed antisolvent (PCA), or solvent, e.g. particle form gas-saturated solution (PGSS) for the preparation of fine particles^24–29^. The RESS is one of the processes in which SCF is used as a solvent. These methods offers several advantages, the most significant being the production of submicron-scale particles that are very small, have the appropriate size and quality distribution, and are also free of solvent. This process consists of two stages. In the first step, the solid soluble component is dissolved in SC-CO_2_ in the saturator or extraction chamber. Subsequently, the sudden expansion of the supercritical solution is done through a nozzle installed at the outlet of the saturating chamber. Due to the rapid decrease in pressure after the solution exits the nozzle, the solubility power is greatly reduced, leading to very fast nucleation and uniform growth of crystals^30–33^. Due to the low solubility of most pharmaceutical compounds in SCF during the RESS process, it will be necessary to use a large volume of SCF to dissolve the required amount of drug, which is another issue with this method. To overcome this problem, a series of processes have been designed to improve the performance of the RESS process. One of the most important methods is the rapid expansion of supercritical solution with cosolvent (RESS-C) process^34,35^. Research findings indicate that when the desired substance does not have proper solubility in the SCF, using the RESS-C method helps prepare particles with nano dimensions. In the RESS-C method, the small particles produced are surrounded by the accompanying solvent, leading to less clumping of the particles and the production of fine particles^36–38^. One study drew on the RESS-SC process to produce Griseofulvin (C_17_H_17_ClO_6_) nanoparticles. The solubility of this medicinal substance in CO_2_ using the solvent with menthol was 28 times higher than its solubility in CO_2_ without using the solvent. Also, by using a nozzle, particles with a diameter of 50–250 nm were produced, which were 12 times smaller than those produced through the RESS process^39^. In another work, the drug Lynestrenol (C_20_H_28_O) was produced by the RESS-SC process on a nanoscale. In this work, the effects of temperature (45–60 °C), pressure (15–30 MPa), and the amount of cosolvent (0–5% w/w) on the final particles were investigated. The results showed that at a temperature of 45 °C and a pressure of 30 MPa, the smallest particle size was obtained in the presence of 5% of menthol, and their average size was less than 100 nm^40^. In another study, the Megestrol acetate (C_24_H_32_O_4_) drug was likewise produced by the RESS-SC process on a nanoscale. In this process, the effect of temperature (40–60 K), pressure (15–25 MPa), and weight percentage of solvent (2–6% w/w) on the final particles was investigated. Increasing the extraction pressure and decreasing the extraction temperature produced finer particles^36^. Below, the articles that have been conducted using the RESS-SC and RESS-C methods to produce fine pharmaceutical particles are listed in Table 1. This research investigates the production of ERL drug nanoparticles using the RESS-C method for the first time. It should be noted that in the previous work, the solubility of erlotinib was measured using SC-CO_2_ in the range of temperature (308–338 K) and pressure (12–30 MPa), which was 0.0045–0.168 g/L. Therefore, in this article, we focused on the production of ERL pharmaceutical nanoparticles to increase the solubility in water with the help of a cosolvent. The study investigated the impact of extraction temperature, pressure, and nozzle diameter on particle diameter and morphology. The effects of temperature (318–338 K), pressure (15–25 MPa), and nozzle diameter (300–700 μm) on ERL particles were investigated using the Box-Behnken design. Finally, the processed particles were characterized using FTIR, DSC, XRD, SEM, and DLS analyses.

**Table 1:** Articles published in relation to RESS-SC & RESS-C method.

Compound	Method	Cosolvent	Parameter		Result	References
Aprepitant	RESS-SC	Menthol	Pressure	15–33 MPa	Particle size: 25.6 μm → 23 ± 1.6 nm	^12^
			Temperature	308.2–338.2 K		
			Nozzle diameter	150–450 μm		
Letrozole	RESS-SC	Menthol	Pressure	12–36 MPa	Particle size: 30 μm → 19 nm	^41^
			Temperature	318.2–348.2 K		
			Spray distance	1–10 cm		
			CoSolvent	1–10 wt.%		
Theophylline	RESS-SC	Vanillin	Pressure	14–22 MPa	Particle size: 71 μm → 85 nm	^42^
			Temperature	265.2–303.2 K		
			Spray distance	3–7 cm		
Tolbutamide	RESS-SC	Menthol	Pressure	150 and 200 bar	Particle size: 89.4 μm → 2.1 μm	^43^
			Temperature	308 and 318 K		
			Nozzle diameter	50 μm		
Griseofulvin	RESS-SC	Menthol	Pressure	95.1–232.2 bar	Particle size: 5 nm → 3000 nm	^39^
			Temperature	35–50 °C		
			CO_2_ density	0.34–0.87 g/ml		
2-aminobenzoic acid	RESS-SC	Menthol	Pressure	96–236 bar	Particle size: 70 μm → 610 nm	^44^
			Temperature	40 and 50 °C		
			Nozzle diameter	100 μm		
Aspirin	RESS-SC	Menthol	Pressure	73–85 bar	Particle size: 479 μm → 6.61 μm	^45^
			Temperature	30–70 °C		
			Spray distance	2–15 mm		
			Nozzle diameter	300–700 μm		
Lynestrenol	RESS-SC	Menthol	Pressure	15–30 MPa	Particle size: 10 μm → 325 nm	^40^
			Temperature	45–60 °C		
			CoSolvent	0–5% w/w		
Megestrol acetate	RESS-SC	Menthol	Pressure	15–25 MPa	Particle size: 8 μm → 516 nm	^36^
			Temperature	40–60 °C		
			CoSolvent	2–6% w/w		
Deferasirox	RESS-C	Acetone	Pressure	140–200 bar	Particle size: 5 μm → 50 nm	^46^
			Temperature	308–318 K		
			Nozzle diameter	500–1200 μm		
Digitoxin	RESS-C	Ethanol	Pressure	10 MPa	Particle size: 8 μm → 458 nm	^47^
			Temperature	90–110 °C		
			Spray distance	3–7 cm		
Naproxen	RESS-C	Methanol	Pressure	200–300 bar	Particle size: 60 μm → 220 nm	^48^
			Temperature	60–100 °C		
Lonidamine	RESS-C	Dichloromethane	Pressure	10–30 MPa	Particle size: 100 μm → 72 nm	^49^
			Temperature	308–328 K		
			CoSolvent	0–5% w/w		
Salicylic acid & Paclitaxel	RESS-C	Ethanol	Pressure	15–25 MPa	Particle size: 300 μm → 1700 nm	^50^
			Temperature	308–333 K		
			Spray distance	6–13 cm		
L-menthol	RESS-C	Ethanol	Pressure	10–20 MPa	Particle size: 400 μm → 1000 nm	^51^
			Temperature	30–50 °C		

## Material and methods

### Materials

**Figure 1:**
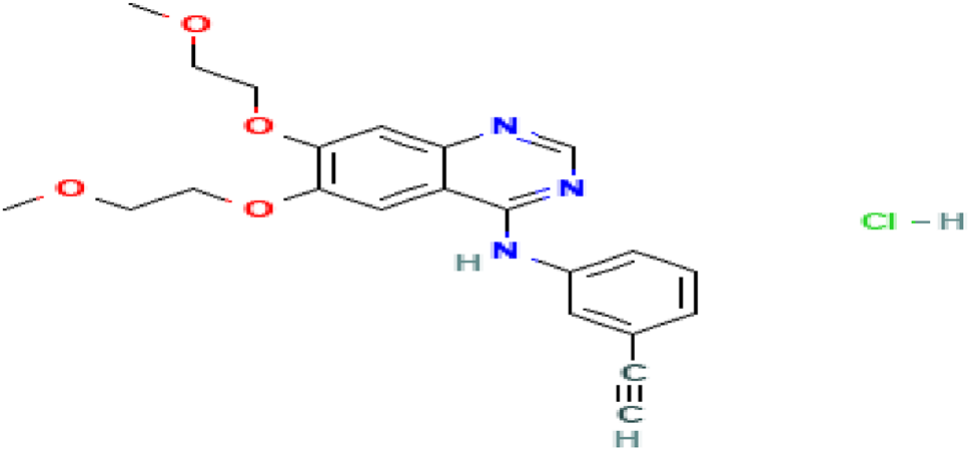
Molecular shape of Erlotinib HCl.

Erlotinib hydrochloride (CAS number. 183319-69-9) with the minimum mass purity of 99.8% was purchased from Parsian pharmaceutical company (Karaj, Iran). Ethanol (CAS number. 64-17-5) with a mass purity of 99.99% was provided by Merck (Darmstadt, Germany), and the carbon dioxide gas (CAS number. 124-38-9) with a mass purity of 99.99% was provided by Zagros Company (Shiraz, Iran). The information of the chemicals used in this project is given in Table 2. Also, the molecular shape of Erlotinib HCl show in Fig. 1.

**Table 2:** The information of the chemicals used in this project.

Compound	Formula	CAS number	Mass fraction purity (%)	Analysis method
Erlotinib HCl	C_22_H_23_N_3_O_4_.HCl	183319-69-9	99.80	HPLC^a^
Ethanol	C_2_H_5_OH	64-17-5	99.99	GC^b^
Carbon dioxide	CO_2_	124-38-9	99.99	GC

^a^High-performance liquid chromatography.

^b^Gas chromatography.

### Methods

#### Design of experiments

The design of experiments (DOE) refers to a set of targeted investigations and controlled experiments that are carried out based on the statistical evaluation of the results to reach answers at a certain level of confidence. Experiment design methods are well-known methods in research and development departments of many industries. While reducing cost and time, these methods provide substantial information about the system or process. Such methods are highly effective in saving money and time when the influencing factors are increased^52^. The response surface method (RSM), for short, refers to the use of mathematical methods and statistical techniques to build experimental models of the studied process. The design of experiments using the RSM was first formed in the 1950s, and its initial application was mainly for the chemical industry. Recently, the RSM has been widely used for quality improvement, product design, and uncertainty analysis^53^. The two most common methods of designing experiments using the RSM are the Central Composite Design (CCD) method and Box-Behnken designs (BBD)^54^. In this study, the values of pressure, temperature, and nozzle diameter were optimized by the experimental design method. For this purpose, 15 experiments were designed with RSM and BBD in Design Expert 7.0.0 Trial software. The three variables of pressure (X_1_), temperature (X_2_), and nozzle diameter (X_3_) were selected at three levels (− 1, 0, + 1), along with three central points representing low, medium, and high values, respectively (Table 3).

**Table 3:** Parameters and their levels for the purpose of Box–Behnken experimental design.

Parameters	Level 1 (− 1)	Level 2 (0)	Level 3 (+ 1)
Pressure (MPa); X_1_	15	20	25
Temperature (K); X_2_	318	328	338
Nozzle diameter (μm); X_3_	300	500	700

#### Apparatus and procedure of RESS

**Figure 2:**
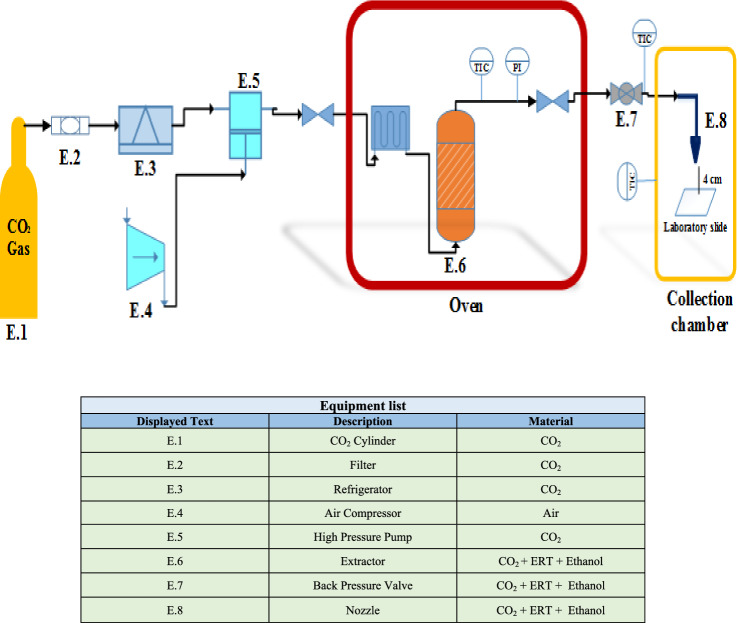
A view of the RESS-C process and equipment list.

An overview of the RESS device and the system components used in this project is illustrated in Fig. 1. The system consists of two parts: extraction and precipitation units. To keep the temperature constant in the extraction cell, the oven temperature was controlled with an accuracy of ± 0.1 K. The equilibrium extraction cell with the volume (70 ml) was placed in a constant temperature oven. The body of the extraction cell, valves, and pipes are made of stainless steel. The pressure of the equilibrium extraction cell was measured by a digital pressure gauge with an accuracy of ± 0.1 MPa. A metal sinter filter was used at the beginning and end of the cell to prevent the overflow of undissolved substances in the SCF flow. After it passes through the refrigerator, the CO_2_ gas purified by the filter (pore size 1 μm) is reduced in temperature (253 K) and subsequently condensed to reach the desired pressure by passing through the high-pressure pump (type-CA 91502, Burbank, CA, USA). The extraction cell was uniformly filled with 1 g of ERL drug and 4 cc of ethanol as a cosolvent. Notably, glass beads with a diameter of 2 mm were used to achieve better dissolution and dispersion of particles. According to Fig. 2, the precipitation unit includes three parts: expansion chamber, nozzle, and nanoparticle powder collector. The primary component of the precipitation unit is the nozzle. During the expansion process, a significant temperature decrease occurs as a result of the Joule–Thomson phenomenon (type MHSS-FL4, FITCA, France). In order to prevent the nozzle from freezing and blocking, a heater was utilized, while a controller monitored and recorded the temperature. When SC-CO_2_ was injected into the extraction cell and heated to the necessary temperature in the oven, the saturated ERT-CO_2_-Ethanol solution was transferred to the nozzle after a particular time (120 min). A Back Pressure Valve (type-1/4FNPT, Xi'an Shelok Instrument Technology Co) is a device for adjusting system pressure and flow control (desired flow rate) with the help of a metering valve. The sample collection tool was placed inside the collection chamber. Inside the collection chamber, the slide was placed on the holding plate^41^. Also, the distance between the slide and the nozzle tip, known as the spray distance, was considered a fixed value of 4 cm. Finally, the fine drug particles sprayed on the slide were collected in the expansion chamber. Then, the samples were prepared for DLS and SEM analyses to check the size and morphology of the particles.

#### Particle characterization

In this project, five analyses, scanning electron microscope (SEM), dynamic light scattering (DLS), Fourier transform infrared (FTIR), X-ray diffraction (XRD), and differential scanning calorimetry (DSC) were used to identify the physical and chemical properties of ERL nanoparticles produced by the RESS-C method. The FTIR device (Tensor II, Bruker Co., Germany) was used to identify molecules and functional groups. DSC (TA Co., USA) analysis is a method to obtain qualitative and quantitative information about the effect of heat on different types of materials. Before DSC analysis, 10 mg of the ERL was placed in a 40 μl aluminum standard pan, and the samples were heated between the ambient temperature and 300 °C at a rate of 10 °C/min. SEM (MIRA III, TESCAN Co., Czech Republic) was used to image the samples, as well as to evaluate the images and obtain the particle size and their distribution. DLS (SZ-100, Horiba Co., Japan) analysis was use to determined the particle size distribution. For this purpose, 1 mg of the ERL (obtained by the RESS-C method) was dissolved in 3 cc of deionized water and placed in a hot water bath at 30 °C for 10 min. XRD (D8 ADVANCE, Bruker Co., Germany) device is an essential tool used for phase identification by comparing the information of known structures. This device was used to test and examine original drug samples and produced ERL nanoparticles by the RESS-C device from an angle of 5° to 80° at a speed of 0.02 degrees per second.

## Results and discussion

In our previous work, the solubility of ERL in SC-CO_2_ was measured in different temperature and pressure ranges (308–338 K and 12–30 MPa)^55^. The present work produced pharmaceutical nanoparticles (ERL) using the REES-C method (15–25 MPa, 318–338 K, and nozzle diameter 300–700 μm). Using the RESS-C method, it was found that the size of the ERL-produced particles decreased from 31.6 μm to 200–1100 nm. Also, the effect of temperature, pressure and nozzle diameter parameters on particle size was investigated. Research findings indicate that reducing the diameter of particles up to 1 μm has minimal impact on solubility. However, when the particle diameter is further reduced, solubility experiences a significant increase. The Freundlich-Ostwald equation^56^ explain why the saturation solubility of nanosuspension increases markedly compared to suspension containing microparticles. Increasing the saturation solubility, dissolution rate, and intrinsic dissolution rate accelerates the dissolution of the drug after consumption. This equation expresses the relationship between solubility and particle diameter as follows:

1 $$\text{log}\frac{\text{Cs}}{\text{C}_\infty }= \frac{2\sigma \text{V}}{2.303\text{RT}\rho \text{r}}$$

In Eq. (1), C_s_ is the solubility of the drug, C_∞_ is the solubility of the solid consisting of large particles, σ is the interfacial tension substance, V is the molar volume of the drug, R is the gas constant, ρ is the average density of the particles, r is the radius, and T is the absolute temperature of the system. Another possible explanation for the increased saturation solubility is the creation of high energy levels during the disruption of more or less ideal drug microcrystals to nanoparticles. According to Ostwald–Freundlich, C_s_ is also a function of the interfacial tension s, which means the interfacial energy G (G = σ ·A). Such differences in interfacial energy are the reason for the differences in C_s_ of polymorphic forms; the same might be valid for the nanosuspension (high energy form = polymorph II = higher C_s_) compared to microparticulate suspensions (low energy form = stable polymorph I = lower C_s_)^57^.

### Effect of different parameters on particle size

**Figure 3:**
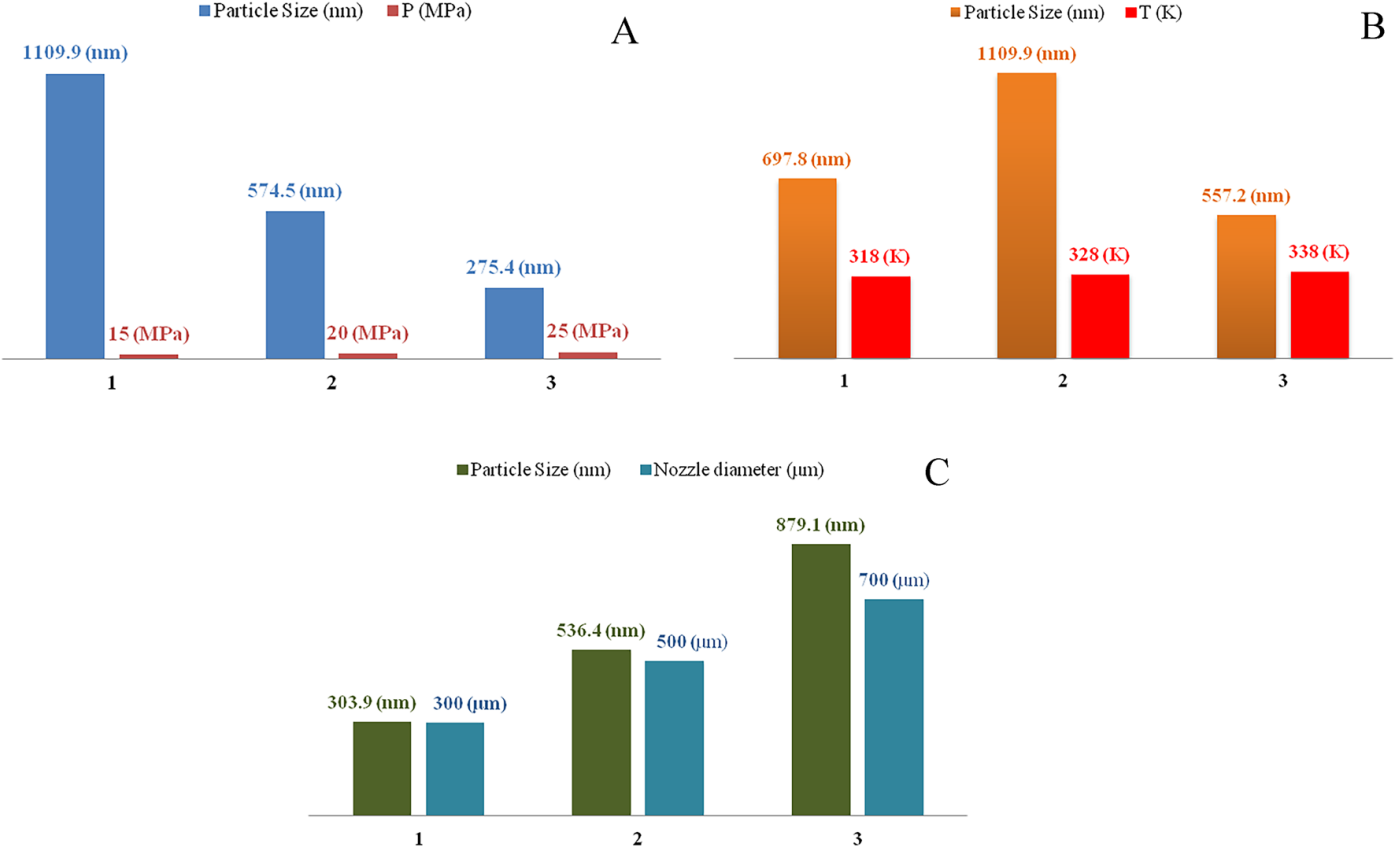
Diagram of the effect of parameters on particle size: (**A**) pressure (at constant temperature 328 K and nozzle diameter 500 μm); (**B**) temperature (at constant pressure 15 MPa and nozzle diameter 500 μm); (**C**) nozzle diameter (at constant pressure 15 MPa and temperature 328 K).

The RESS-C method was employed to investigate the effect of different parameters on the size of the produced particles. The results (Fig. 3) revealed the parameters, namely temperature (318–338 K), pressure (15–25 MPa), and nozzle diameter (300–700 μm), were among the most important parameters affecting these processes. BBD (L-15) method was used to design the effect of RESS-C process parameters on the diameter and structure of produced ERL particles. One of the most important advantages of the BBD method for test design is the small number of tests, which is up to 3 factors. With this method, many factors can be designed with fewer tests^58,59^. This work investigated the impact of three influencing parameters (temperature, pressure, and nozzle diameter) on the size of produced particles. The main parameters were selected based on various experiments. The number of tests based on the BBD is given in Table 4.

**Table 4:** The number of runs based on the BBD method.

Run	T (K)	P (MPa)	Nozzel diameter (μm)	Mean particle size (x_50_—nm)	Predicted value (nm)
1	328	25	700	697.8	704.9
2	338	20	300	255.9	211.2
3	328	20	500	557.2	601.9
4	328	15	700	421.8	414.8
5	328	25	300	528.5	499.7
6	318	15	500	275.4	298.2
7	338	20	700	1110	1087.1
8	318	25	500	578.8	607.7
9	318	20	300	336.8	358.6
10	338	25	500	303.9	288.1
11	318	20	700	670.3	686.2
12	338	15	500	879.1	857.3
13	328	15	300	574.5	548.2
14	328	20	500	533.7	548.2
15	328	20	500	536.4	548.2

Based on the initial tests and existing limitations for the production of ERL nanoparticles by the RESS-C process, the temperature and pressure conditions in the extraction tank (15–25 MPa and 318–338 K) were selected. According to the ANOVA results shown in Table 5, the quadratic model was suggested among the (linear, two-factor interaction (2FI) and Cubic) models for the production of ERL nanoparticles. Also, the number of mentioned parameters and their importance for the production of ERL nanoparticles are given in Table 6. According to Table 6 and the (P-value) values obtained, the primary parameters (temperature, pressure, and nozzle diameter) significantly affected the production of ERL nanoparticles. The impact of each parameter was evaluated according to the values (P-values). Any parameter with P-values less than 0.05 is known to have a significant effect on the process with 95% confidence, and parameters with P-values greater than 0.05 have little effect on the process^41,60,61^. Therefore, according to Table 6, temperature, pressure, and nozzle diameter had a significant impact. Moreover, the F-values in Table 5 show that the parameters of temperature, pressure, and nozzle diameter are effective in the process. In addition, the values of the determination coefficient, R^2^ (R-square), adjusted R^2^, and predicted R^2^ were 0.9875, 0.9650, and 0.8194, respectively. Therefore, according to the obtained results, it is clear that the method used is acceptable.

**Table 5:** ANOVA results.

Source	Std. Dev	R-square	Adjusted R-square	Predicted R-square	Sequential P-Value	Lack of fit P- value	PRESS
Linear	98.18	0.8577	0.8189	0.6966	< 0.0001	0.0434	2.260E + 005
2FI	77.95	0.9348	0.8858	0.6585	0.0864	0.0629	2.544E + 005
Quadratic	43.14	0.9875	0.9650	0.8194	0.0303	0.1631	1.346E + 005
Cubic	22.82	0.9986	0.9902		0.1631

**Table 6:** ANOVA analysis of ERL nonoparticles.

Source	Sum of squares	df	Mean square	F value	P-Value	Significance
model	7.357E + 005	9	81,744.37	43.92	0.0003	Significance
A = Pressure	2.318E + 005	1	2.318E + 005	124.53	0.0001	Significance
B = Temperature	5059.12	1	5059.12	2.72	0.0160	Significance
C = Nozzle diameter	4.022E + 005	1	4.022E + 005	216.10	< 0.0001	Significance
AB	23,500.74	1	23,500.74	12.63	0.0163	Significance
AC	19,316.14	1	19,316.14	10.38	0.0234	Significance
BC	14,603.27	1	14,603.27	7.85	0.0379	Significance
A^2^	104.32	1	104.32	0.056	0.8222	Not Significance
B^2^	18,252.78	1	18,252.78	9.81	0.0259	Significance
C^2^	17,930.23	1	17,930.23	9.63	0.0267	Significance
Residual	9305.01	5	1861.00			
Lack Of Fit	8263.60	3	2754.53	5.29	0.1631	Not significance
Pure Error	1041.41	2	520.70			
Cor Total	7.450E + 005	14				

**Figure 4:**
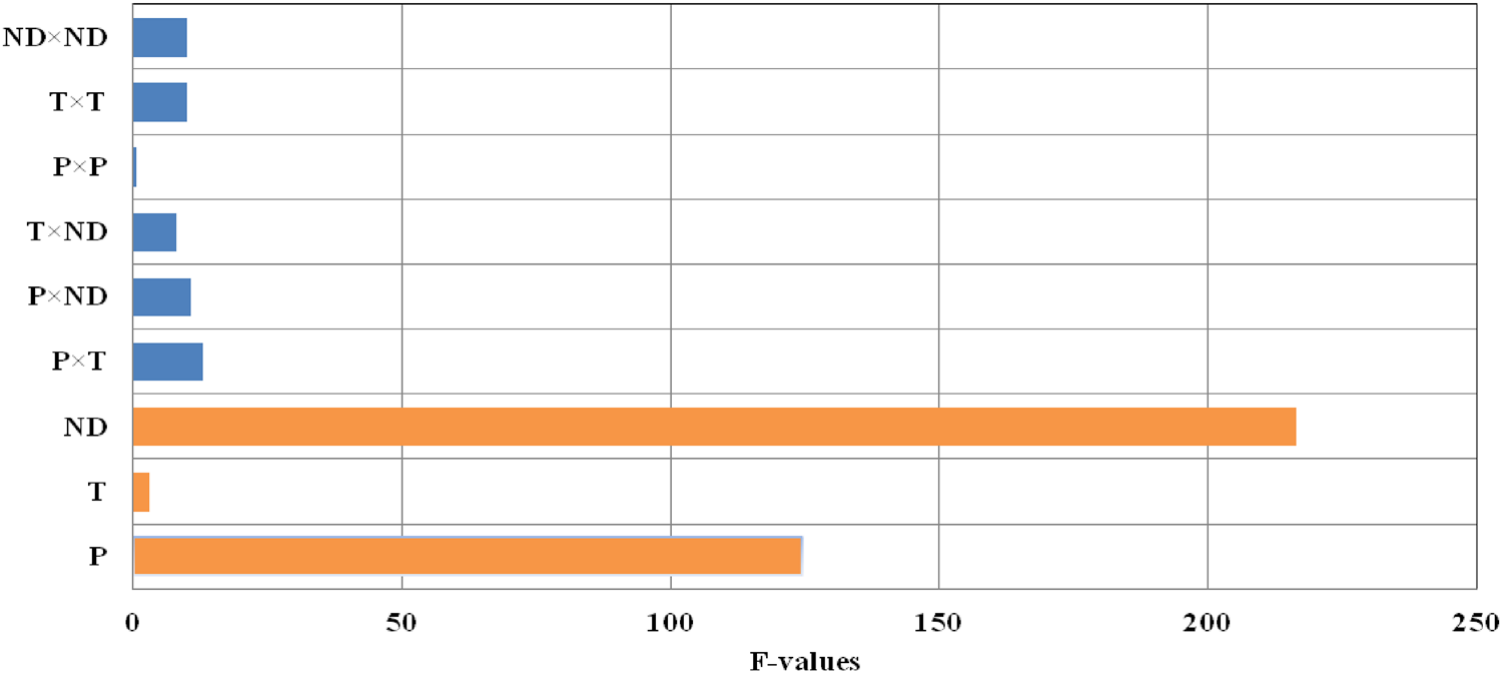
Pareto chart for effective parameters in BBD method.

According to Fig. 4, the factors affecting the particle size were analyzed by the Pareto chart, and it was found that the nozzle diameter (ND) (P-value =  < 0.0001) has the most significant effect on the particle size. Also, the parameters of pressure (P) (P-value = 0.0001) and temperature (T) (P-value = 0.0160) are other factors affecting the size of the produced particles.

#### Effect of pressure

As shown in Fig. 3a, the diameter of precipitated particles decreases with increasing pressure from 15 to 25 MPa in this study. On the other hand, increasing the pressure enhances the solubility of the material, and as a result, the supersaturated state is better in the expansion zone. Therefore, according to the classical theory, the nucleation rate is higher, and finer particles are produced. Hence, with the increase in solubility and the production of finer particles, the growth rate of particles in the expansion chamber increases due to the large number of particles^62,63^. In this study, as the pressure increased, the ERL concentration increased. Moreover, a higher supersaturation and nucleation rate was obtained at the nozzle tip, leading to the formation of smaller particle sizes. It was found that the effect of pressure increase on the nucleation and growth rates has been overcome. Similar results have been observed in the articles by Sodeifian et al.^64^, Esfandiari et al.^65^, Honarvar et al.^19^, and Reverchon et al.^66^ regarding the effects of pressure and temperature on particle diameter. It is worth mentioning that Hezave et al.^67,68^ observed different results regarding the effect of temperature and pressure when micronizing Fenoprofen (C_15_H_14_O_3_) and Ketoprofen (C_16_H_14_O_3_) drug particles.

#### Effect of temperature

Based on Fig. 3b, the effect of temperature on the production of nanoparticles was investigated in the range 318–338 K. It should be noted that the spraying distance (4 cm) and nozzle diameter (300 μm) were considered constant. In general, temperature has a dual effect on particle size. Increasing the temperature also had a different effect on the particle size. Increasing the extraction temperature decreased the density of CO_2_, thereby increasing the vapor pressure of the dissolved substance in CO_2_. As a result, the decrease in density causes a decrease in solubility, and an increase in vapor pressure causes an increase in solubility^67,69,70^. In this research, larger particles were formed with increasing temperature. Similar results were observed by Asghari et al.^71^ and Turk et al.^72^ for producing carboxymethyl cellulose (C_8_H_16_O_8_) and phytosterol (C_29_H_50_O) nanoparticles. The increase in temperature causes a decrease in the density of CO_2_, thus decreasing its solubility and creating a lower supersaturation state in the chamber after expansion. This causes a lower nucleation rate and the production of larger particles^73,74^. In this case, the impact of temperature on density has prevailed over its effect on vapor pressure. According to Fig. 3b, the smallest and largest particles were created at 338 and 328 K, respectively.

#### Effect of the nozzle diameter

The results about the effect of increasing the diameter of the nozzle showed that by increasing the diameter of the nozzle from 300 to 700 μm, there was an increasing trend in the average diameter of the produced particles (Fig. 3c). The reason for this situation can be interpreted as follows: when the diameter of the nozzle is smaller, the expansion time of the particles becomes shorter. As a result, the nucleation rate increases, leading to the formation of smaller particles at a faster rate^75^. Of course, as mentioned earlier, an increase in the nucleation rate can increase the rapid growth of particles due to the large number of particles. Nonetheless, in this work, the effect of nucleation speed has overcome the speed of particle growth^12,76^.

### ERL characterization

#### SEM

**Figure 5:**
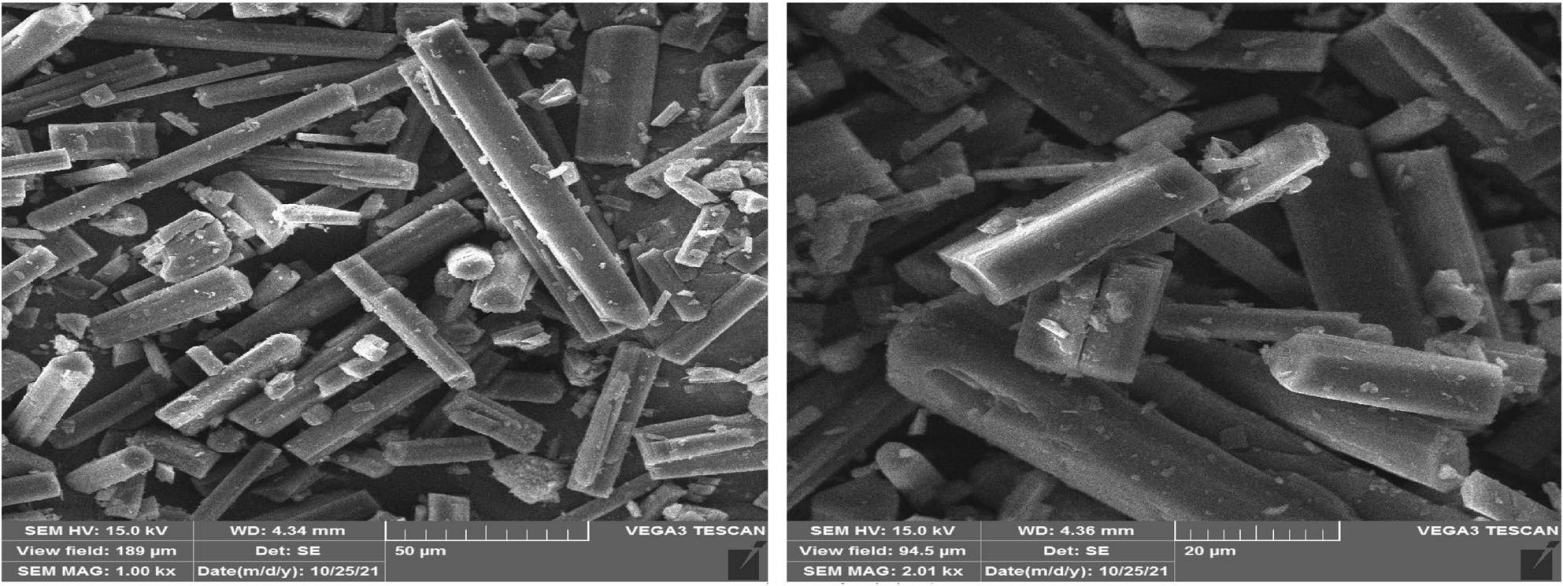
SEM image of original ERL.

**Figure 6:**
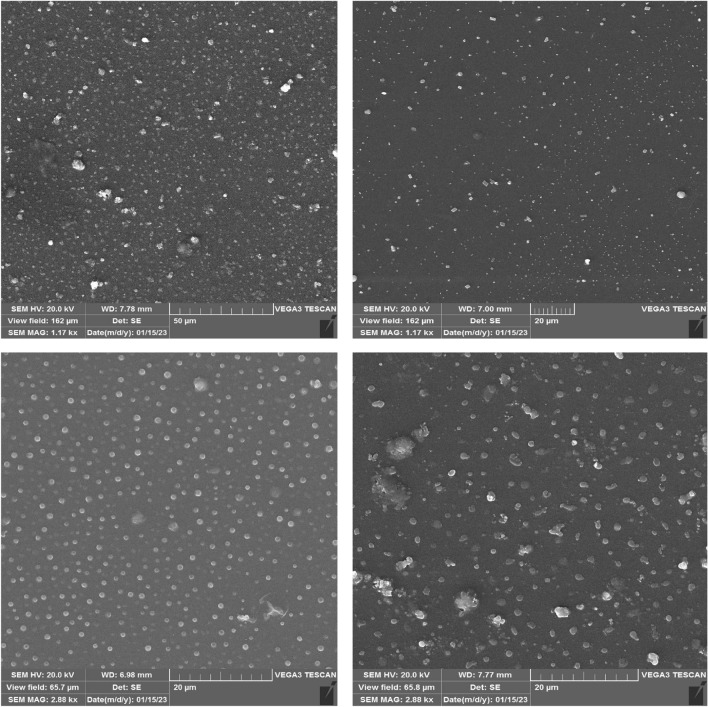
SEM images (according to Table 4): (**A**) Run 7 (**B**) Run 3 (**C**) Run 10 (**D**) Run 6.

Figure 5 depicts the SEM image of the original ERL with particle size distribution. According to Fig. 5, it is found that the original ERL particles have a diameter of about 31.60 μm and a rod shape. Following the results, SEM images of some ERL samples processed with RESS-C are depicted Fig. 6a–d. The average size of the obtained particles is 550 nm, and their shape is spherical.

#### DLS

**Figure 7:**
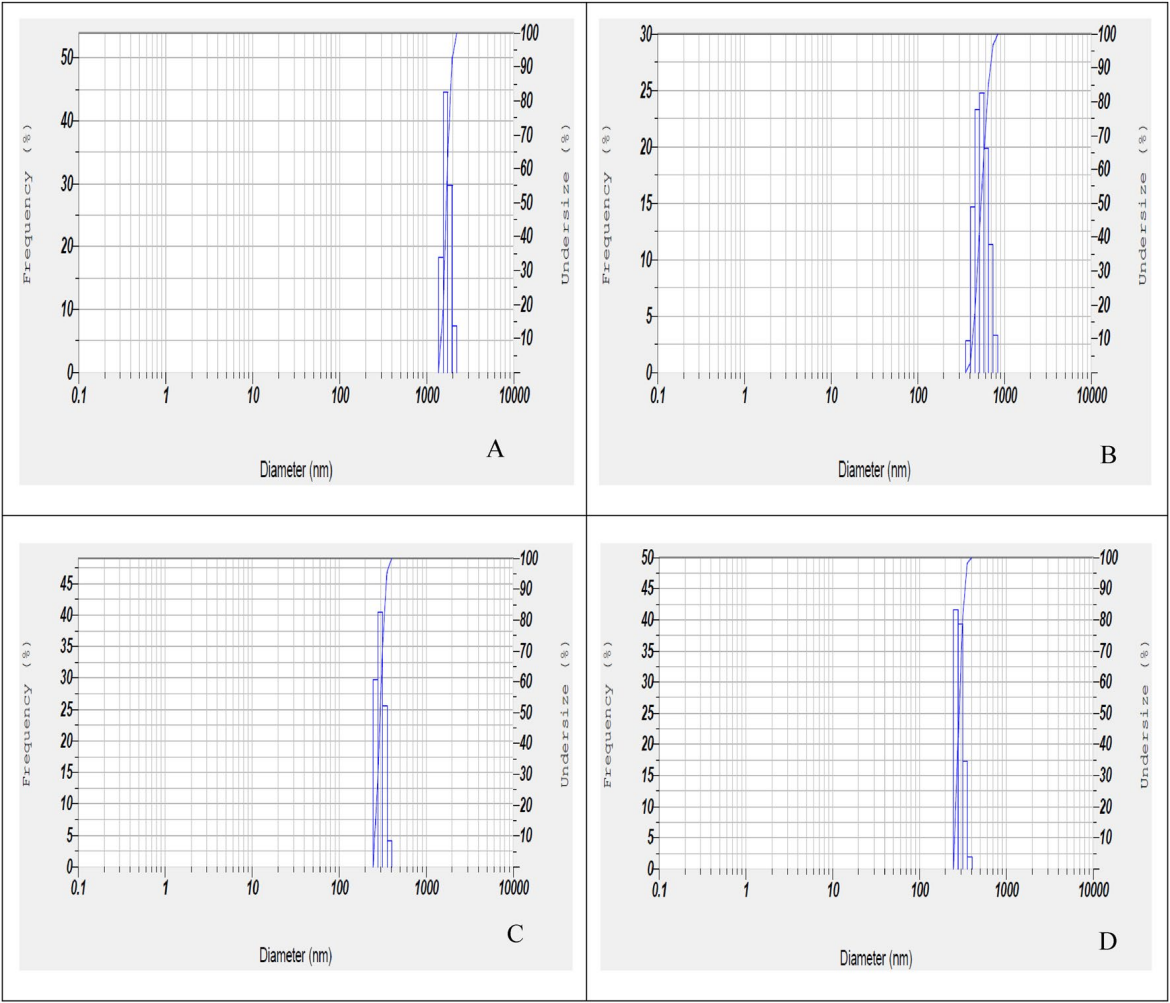
DLS results (according to Table 4): (**A**) Run 7 (**B**) Run 3 (**C**) Run 10 (**D**) Run 6.

DLS tests were performed under different temperature, pressure, and nozzle diameter conditions, according to Table 4. Also, DLS results are reported in Fig. 7a–d (according to Table 4): (A) Run 7, (B) Run 3, (C) Run 10, and (D) Run 6, based on SEM images (Fig. 6a–d).

#### FTIR

**Figure 8:**
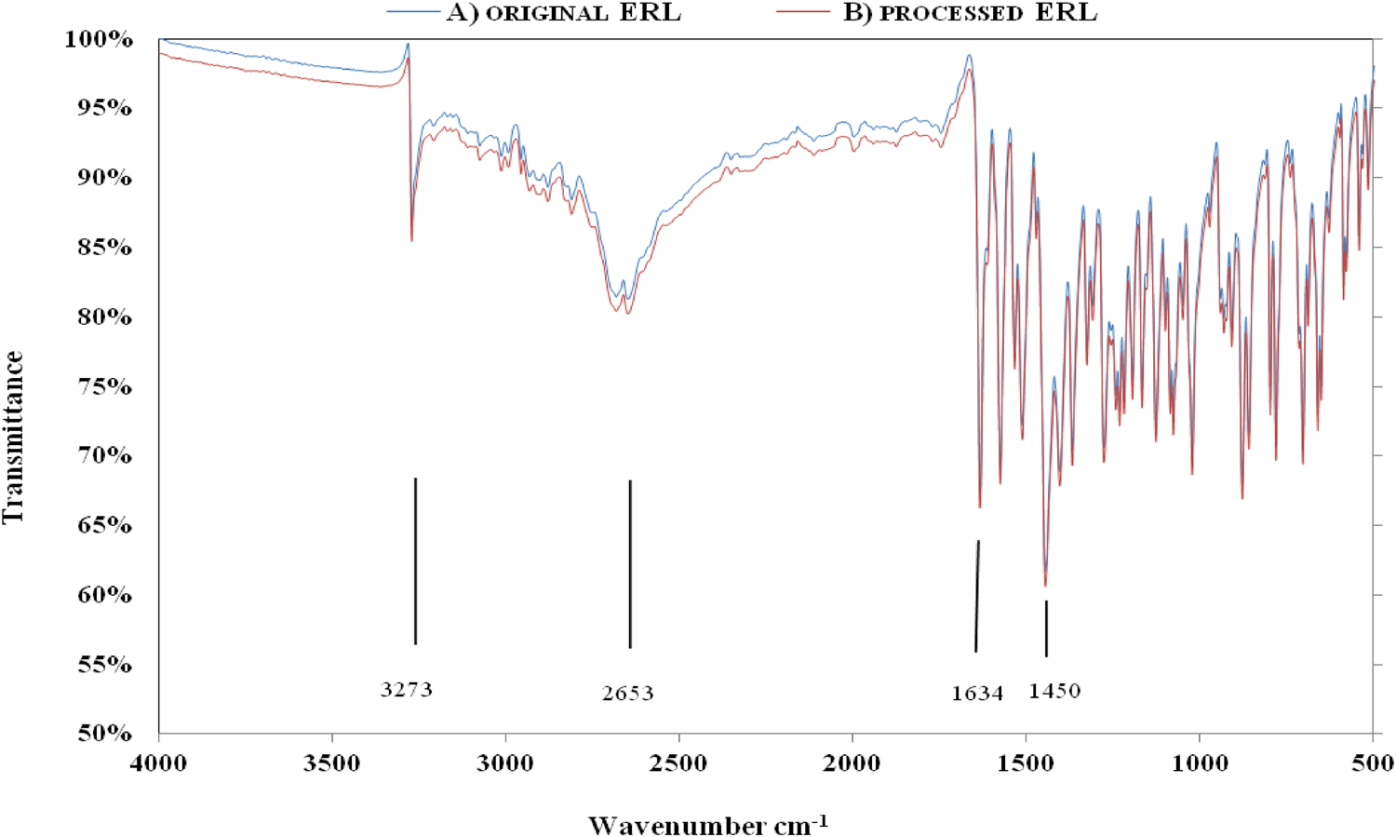
FTIR analysis of (**A**) original ERL (**B**) processed ERL.

It was used to investigate the chemical structure of organic compounds. Since the pressure in the RESS process is very high, it is imperative to examine the possibility of changing the chemical structure of ERL under the influence of this process, especially since this compound is supposed to be used in pharmaceutical formulations. The FTIR device provides the spectra of compounds in the 400 cm^-1^ to 4000 cm^-1^ (2.5 to 25 μm) with a resolution of 2/cm. Jahangiri et al.^77^, Piergies et al.^78^, and Tabtimmai et al.^79^ measured the infrared spectrum of ERL at wavelengths 400 to 4000/cm. Figure 8a illustrates the FTIR spectrum of the original ERL. One of the prominent peaks of the original ERL is related to the stretching bond =NH–, which can be seen in wave number 3273. The ≡C–H stretching bond is one of the other prominent peaks in the infrared spectrum of ERL, observed at the wavelength of 2653. The bending NH and stretching –C–N bonds are observed in wave numbers 1634 and 1450, respectively. As shown in Fig. 8b, the prominenet peaks of processed ERL nanoparticles are observed at wave numbers 3270 (stretching bond =NH–), 2651 (stretching bond ≡C–H), 1630 (bending bond NH), and 1447 (stretching bond –C–N). The interpretation of FTIR spectra is given in Table 7. Therefore, the results show that the chemical structure of ERL was not modified during the RESS-C process. It is important to mention that the changes in the location of functional groups in the chemical structure of ERL result from various factors. These factors include the shift in the frequency of oscillations caused by the reduction of particle size, the interactions of dipole attraction or repulsion, and the presence of an amorphous surface on nanoparticles^80^.

**Table 7:** FTIR spectrum for original ERL and nanoparticles.

Chemical functional bond	Original ERL	ERL nanoparticles
stretching bond =NH–	3273	3270
stretching bond ≡C–H	2653	2651
bending bond NH	1634	1630
stretching bond –C–N	1450	1447

#### XRD

**Figure 9:**
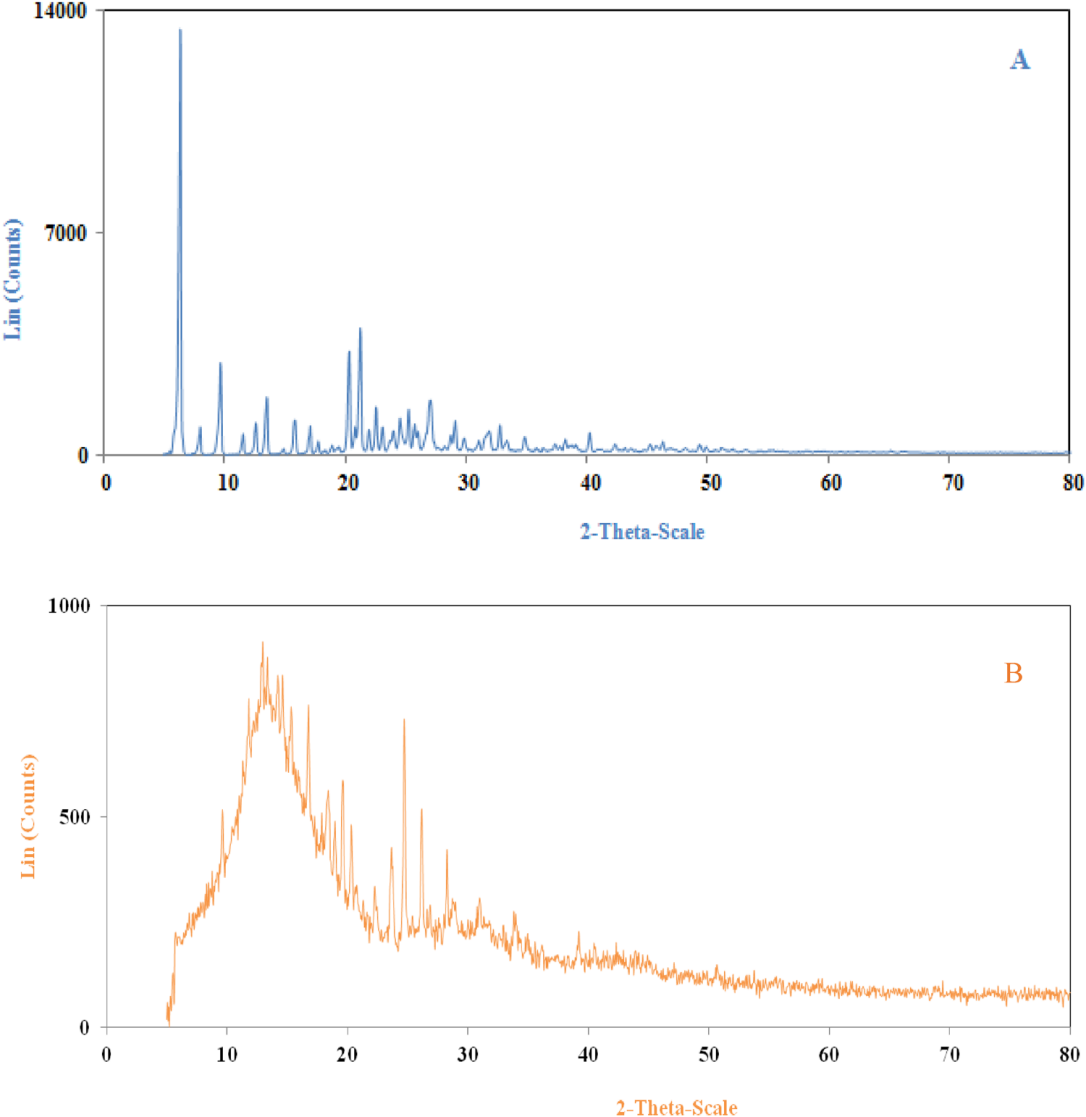
XRD spectrum of (**A**) original ERL and (**B**) processed ERL.

It was used to identify materials with a crystalline structure. The presence of new peaks, a change in the location of the peaks, or a shoulder of the peaks can be proof of the presence of a new polymorph. The XRD spectrum of the original ERL is depicted in Fig. 9. As can be seen, the original ERL has several indicator peaks at angles of 6.38, 9.73, 13.53, 20.36, and 21.23. The presence of these peaks indicates the crystalline structure of ERL. Also, in Fig. 9. ERL nanoparticles have several indicator peaks in the same positions as the original ERL. From the comparison of these two graphs, it is clear that there have been slight changes in the location of the peaks. However, the intensity of the peaks has significantly been reduced. This decrease in the intensity of the peaks can have two reasons. (A) The decrease in the degree of crystallinity of ERL nanoparticles. (B) The particle size reduction from the micrometer to the nanometer scale. SEM images (Fig. 5) show a drastic reduction in particle size. Also, the comparison of the diffraction spectra of the original ERL and nanoparticles shows that the RESS-C process did not change the crystal structure of ERL. Similar results have been reported by other researchers, including Tien et al.^81^, Abdelgalil et al.^82^, Zhai et al.^83^, and Pandey et al.^84^.

#### DSC

**Figure 10:**
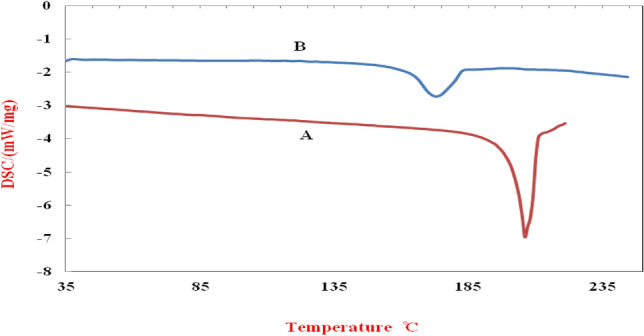
DSC curve of (**A**) original ERL (**B**) processed ERL.

The polymorph type of the crystal structure, thermal behavior, and degree of crystallinity were used to determine the melting point. Figure 10a,b shows the thermal behavior (DSC graph) of the original ERL and its nanoparticles (heat flow in terms of temperature). As shown in Fig. 10a, the melting point peak of the original ERL is at 229.4 °C. In the research conducted by Truong et al.^85^ and Jahangiri et al.^77^, the same melting point as the present research has been reported. The thermal behavior of ERL nanoparticles, as depicted in Fig. 10b, indicates a reduction in the melting point by 56.5 °C when compared to the original ERL. However, it exhibits a comparable thermal behavior to the original ERL. The reduced melting point can be a reason for the decrease in nanoparticles' crystallinity degree, confirming the XRD results. The network with low crystallinity or amorphy in the melting process requires less energy to overcome the network forces than the material with an ideal crystal structure. To measure the amount of crystal identity (CI), the amount of energy consumed for melting can be used. Therefore, this parameter can be calculated using the following relation^86^.

2 $$\text{\% CI}=\frac{{\Delta \text{H}}_{2}}{{\Delta \text{H}}_{1}}\times 100$$

In this equation, ∆H_1_ is the amount of normalized energy change in the melting process for the original ERL, and ∆H_2_ is the amount of normalized energy change in the melting process for ERL nanoparticles (Table 8). According to the normalized energy change values in Table 8, the % CI is 63. Another reason for the decrease in melting point is the drastic reduction in particle size. The reason for this is that due to the decrease in the size of the particles, the level of heat transfer has increased, leading to a reduction in the melting point of ERL nanoparticles^87^.

**Table 8:** Values of melting temperature and enthalpy change of original ERL and nanoparticles.

Item	The enthalpy changes ΔH(j/g)	Onset (°C)	Peak (°C)	End set (°C)
Original ERL	127.6	225.8	229.4	233.8
ERL nanoparticle	80.39	164.0	172.9	182.7

## Conclusion

In this research, the production of ERL medicinal nanoparticles by SCF has been investigated, considering the advantages of using this fluid type. The research results showed that the RESS-C process is one of the standard processes in which SCF is used as a solvent to produce fine particles. Next, the effect of different parameters on the size of the produced particles was investigated, and the results showed that parameters such as temperature (318–338 K), pressure (15–25 MPa), and nozzle diameter (300–700 μm) were among the most important parameters affecting these processes. Since little research has been conducted in this area, the ERL nanoparticles produced by the RESS-C process were investigated in detail. Using the RESS-C method, it was found that the size of the ERL-produced particles decreased from 31.6 μm to 200–1100 nm. By examining the FTIR spectrum for manufactured pharmaceutical nanoparticles, it was found that the chemical structure of the particles did not change during this process (high pressure). XRD of ERL nanoparticles shows that the crystalline structure of nanoparticles has been preserved, and no changes have been made. The results of the DSC of nanoparticles show that the melting point of 56.5 °C has decreased compared to the original ERL, although their thermal behaviors are similar.

## Data Availability

All data generated or analysed during this study are included in this published article.
